# A conserved acidic patch in the Myb domain is required for activation of an endogenous target gene and for chromatin binding

**DOI:** 10.1186/1476-4598-7-77

**Published:** 2008-10-07

**Authors:** Emily Ray Ko, Dennis Ko, Carolyn Chen, Joseph S Lipsick

**Affiliations:** 1 Departments of Pathology and Genetics, Stanford University School of Medicine, Stanford, CA 94305-5324, USA

## Abstract

The c-Myb protein is a transcriptional regulator initially identified by homology to the v-Myb oncoprotein, and has since been implicated in human cancer. The most highly conserved portion of the c-Myb protein is the DNA-binding domain which consists of three imperfect repeats. Many other proteins contain one or more Myb-related domains, including a number of proteins that do not bind directly to DNA. We performed a phylogenetic analysis of diverse classes of Myb-related domains and discovered a highly conserved patch of acidic residues common to all Myb-related domains. These acidic residues are positioned in the first of three alpha-helices within each of the three repeats that comprise the c-Myb DNA-binding domain. Interestingly, these conserved acidic residues are present on a surface of the protein which is distinct from that which binds to DNA. Alanine mutagenesis revealed that the acidic patch of the third c-Myb repeat is essential for transcriptional activity, but neither for nuclear localization nor DNA-binding. Instead, these acidic residues are required for efficient chromatin binding and interaction with the histone H4 N-terminal tail.

## Background

In vertebrates, c-*Myb *(*MYB*), A-*Myb *(*MYBL1*), and B-*Myb *(*MYBL2*) comprise the *Myb *gene family. Myb proteins encoded by these genes serve essential roles in transcriptional regulation and cell cycle control (reviewed in [1]). Studies of c-*Myb *null and hypomorphic mutant mice have delineated an essential role in hematopoiesis and in the maintenance of intestinal epithelium [2,3]. A-*Myb *null mutant mice display abnormal spermatogenesis and mammary gland proliferation [4]. B-*Myb *null mice suffer from embryonic lethality at the 8-cell stage, suggesting an essential role for B-Myb in cell proliferation that is consistent with the expression of B-*Myb *in all proliferating cell types [5]. Importantly, aberrant expression of these *Myb *genes occurs in a variety of human malignancies including leukemias, lymphomas, breast, colon, brain, pancreatic, and lung cancers (reviewed in [1]).

Proteins in the Myb family share conservation of both an N-terminal DNA-binding domain (DBD) and a C-terminal regulatory domain. The Myb DBD is composed of three imperfect helix-turn-helix repeats and Myb-related proteins have one or more of these Myb-like repeats [6]. In plants, Myb-related repeats have been utilized in hundreds of DNA-binding transcription factors [7]. Other Myb-related proteins serve an assortment of nuclear functions, including chromatin remodeling (SWI3, RSC, ISWI), covalent histone modification (ADA2, NCoR), basal transcription (TFIIIB-B', SNAPC4), transcriptional silencing (REB1, TTF1, RAP1), telomere regulation (TAZ1, TRF1, TRF2, RAP1), and RNA splicing (CDC5). Surprisingly, some of these proteins have conserved Myb-like repeats, but do not utilize this domain to bind DNA [1,6]. Deletion and mutational analysis of such Myb-related repeats has implicated these domains in histone binding [8-11].

While it is tempting to think of DNA-binding domains (DBDs) of proteins as specialized building blocks with a singular DNA-binding function, the multifunctional nature of such domains has previously been reported (reviewed in [12]). In this regard, the distantly Myb-related RAP1 DBD also has nucleosome binding activity and the capacity to alter chromatin structure [13]. Remarkably, although deletion of the entire yeast *RAP1 *gene was lethal, the expression of the Myb-related RAP1 DBD alone was sufficient to restore viability [14]. These results imply that the RAP1 DBD has critical function(s) in addition to simply binding to DNA.

Previous studies have provided evidence for non-DNA-binding functions of the vertebrate Myb DBD, as well. For example, insertional mutations or a cysteine-to-serine substitution within the v-Myb DBD disrupt transcriptional activation and oncogenic transformation without affecting nuclear localization or DNA-binding [15,16]. Consistent with these observations, the Myb DBD has been reported to specifically interact with the DBD of the C/EBPα transcriptional activator, the DBD of the c-Maf protein, and with Cyclin D [17-20].

Intrigued by the presence of Myb-related repeats in so many nuclear proteins, especially those lacking specific DNA-binding capacity, we reasoned that residues conserved among this larger group of Myb-related proteins might uncover novel functions for the c-Myb DBD. By performing alignments of Myb-related repeats of both DNA-binding and non-DNA-binding proteins, we discovered patches of highly conserved acidic residues. Alanine mutagenesis of these residues in each c-Myb repeat identified an important role in transcriptional activation for the acidic patch in the third c-Myb repeat independent of nuclear localization or DNA-binding. Rather, the acidic motif in the c-Myb third repeat was required for chromatin association *in vivo *and for binding to the histone H4 tail *in vitro*. Furthermore, these data suggest that the acidic patches of Myb-related repeats in other proteins may also function in chromatin binding and/or histone association.

## Results

### Myb-related domains contain a conserved acidic patch

**Figure 1:**
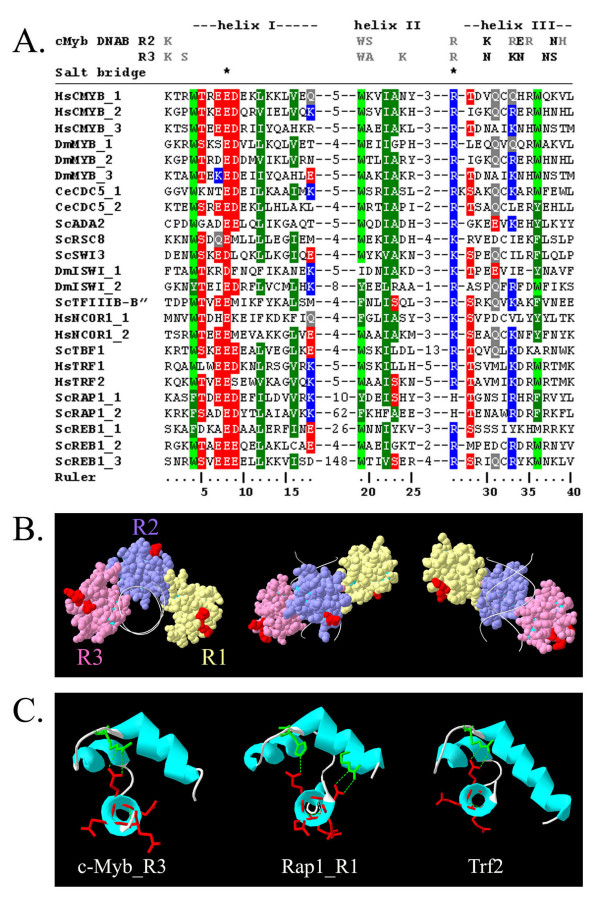
**Amino acid alignments of Myb-related repeats display high conservation of structural residues and an acidic patch in the first helix.** A. We created a multiple sequence alignment of Myb repeats using ClustalW and color coded the alignments based on conservation using the BioEdit program. Labeling of Myb-repeats indicates genus and species with the first two letters, followed by the protein name. For proteins containing multiple Myb-motifs, repeats are numbered starting from the N-terminus and identified after an underscore. Residues known to bind DNA of c-Myb (c-Myb DNAB) second repeat (R2) and third repeat (R3) are depicted in bold on the first two lines, those interacting with DNA bases are in black and those associating with the phosphate backbone are in grey. Note the high conservation of acidic residues in the first helix compared to c-Myb DNA-binding residues. Species and genus abbreviations: Hs = Homo sapiens, Dm = Drosophila melanogaster, Ce = Caenorhabditis elegans, Sc = Saccharomyces cerevisiae. B. The crystal structure of the c-Myb DNA-binding domain bound to DNA is shown [18]. The first repeat (R1) is depicted in yellow, the second repeat (R2) in purple, and the third repeat (R3) in pink. The conserved acidic patch in the first helix of each repeat is highlighted in red, revealing a location on the solution side of the protein consistent with a protein-protein interaction motif. C. Myb-repeats from the solved c-Myb third repeat (c-Myb_R3) [18], yeast Rap1 first repeat (Rap1_R1) [22], and human Trf2 [23]. For each repeat, one is looking down the barrel of the first helix (bottom) with the second helix on the left and the third helix on the right. The conserved acidic residues are depicted in red. Conserved salt bridges are shown between the acidic residue in the first helix (red) and a basic residue in the third helix (green).

Myb-related repeats are conserved in a variety of proteins with very different nuclear functions. Whether the duplication and retention of many genes encoding Myb-related repeats occurred due to the presence of a versatile structural scaffold or whether the preservation of this domain took place due to a conserved biochemical function remains unknown. To determine if conservation of non-structural residues occurred within the diverse array of Myb-related proteins, we created and analyzed alignments of the amino acid sequences of Myb repeats from all known Myb-related protein families. Each Myb-related repeat contains three highly conserved helices connected by two turns/loops that are variable in both sequence and length [6]. Solved structures of several Myb-related domains have revealed that the length, orientation, and spatial organization of the three helices are very similar to the structure of the c-Myb repeats [11,21-23]. Therefore, to align Myb-related repeats from many different proteins we deleted the turn/loop regions and aligned the helices using the ClustalX program [24]. The residues most highly conserved among all Myb-related proteins are those involved in forming the hydrophobic core (tryptophan residues at position four, nineteen, and thirty-six in Figure 1A), indicating that structural integrity of the domain is essential for both DNA-binding and non-DNA-binding Myb repeat functions. Consistent with this structural preservation is the presence of a highly conserved salt bridge between an acidic residue at position eight and a basic residue at position twenty-six (asterisks in Figure 1A).

Aside from residues essential for structural integrity, the most conserved residues among all Myb domains are a stretch of acidic amino acids near the beginning of the first helix (shown in red in helix I, Figure 1A). Remarkably, these acidic amino acids are more highly conserved than residues involved in DNA binding. Glutamic/aspartic acid residues are often present at positions six through ten, but the highest concentration of acidic residues occurs consistently at positions seven through nine. This acidic patch includes the highly conserved salt bridge at position eight, but the conserved adjacent acidic residues do not have a known role in structural integrity or DNA binding.

DNA-binding domains typically exhibit a strongly basic character in order to form ionic bonds with the phosphate backbone of the DNA double helix, making the presence of a patch of highly conserved acidic residues in the Myb DBD rather unusual. Because these acidic residues are also present in non-DNA-binding Myb-related repeats, we hypothesized that these residues might be important for protein-protein rather than protein-DNA interactions. Consistent with such a model is the localization of these conserved acidic residues on a surface facing away from the DNA within the c-Myb crystal structure (shown in red in Figure 1B) [18]. The positions of these acidic patches are conserved in the structures of the distantly Myb-related RAP1 and TRF2 proteins; once again these residues face away from the DNA (Figure 1C) [22,23].

### A conserved acidic patch is required for transcriptional activation by c-Myb

**Figure 2:**
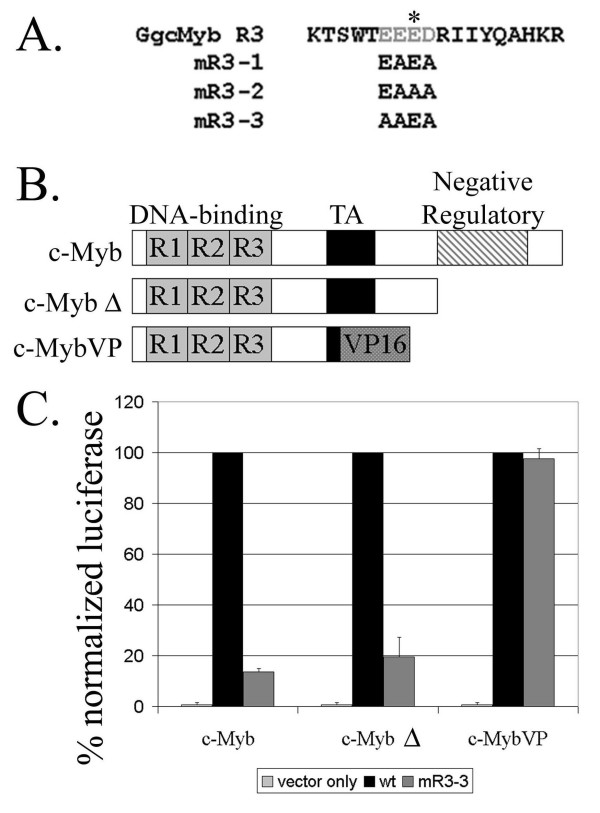
**The mR3-3 is defective in transcriptional activation and this phenotype can be rescued by VP16 fusion to the mutant DNA-binding domain.** A. Alanine mutagenesis of the third c-Myb repeat was performed to analyze the structural and functional contributions of the acidic patch in the first helix of each repeat. Three mutants of the third repeat (mR3-1,2,3) were constructed: mR3-1 preserved the salt bridge and converted two acidic residues to alanines, mR3-2 eliminated the salt bridge, and mR3-3 preserved the salt bridge and changed all three acidic residues not involved in salt bridges to alanines. B. The diagram identifies the positions of the DNA-binding domain (DBD), the transcriptional activation domain (TA), and the C-terminal negative regulatory domain. The DNA-binding domain consists of three imperfect repeats labeled R1, R2, and R3. The truncated form of c-Myb (c-MybΔ) is missing the negative regulatory domain. c-MybVP represents the c-Myb DBD fused to the VP16 activation domain. C. Vector only (v/o), wild type (wt) c-Myb, or the mR3-3 c-Myb mutant constructs in (A) were tested for transcriptional activation of the EW5-E1b-Luciferase reporter. Luciferase activity for each of the mutant c-Myb constructs was normalized for transfection efficiency by measuring β-galactosidase activity from a cotransfected CMV-β-galactasidase plasmid. The wild-type c-Myb values were then set to 100% for each class of proteins and the mutants were scaled accordingly, allowing the comparison of multiple experiments and comparison of c-Myb, c-MybΔ, and c-MybVP. Before normalization the wild-type c-MybΔ and c-MybVP had a transcriptional activity 100× and 1000× greater than the full length c-Myb, respectively.

To explore the function of these highly conserved acidic residues, we performed alanine mutagenesis of the acidic patch in each of the three repeats of chicken c-Myb. Mutant proteins were analyzed for expression, stability, nuclear localization, DNA-binding, and transcriptional activity. Table 1 summarizes the results of these assays for mutants in all three c-Myb repeats. In particular, we tested the ability of each mutant to transcriptionally activate a luciferase reporter regulated by five tandem high-affinity Myb-binding sites (A site) from the *mim-1 *target gene placed upstream of a minimal E1b TATA box (EW5-E1b-Luciferase reporter) [25,26]. This reporter was co-transfected with wild-type or mutant c-Myb expression plasmids into the QT6 quail fibroblast cell line. These cells have the advantages of having no endogenous c-Myb expression and of being highly transfectable. We found that mutagenesis of acidic residues without a known role in structural stability (i.e. participation in salt bridges) did not disrupt transcriptional activity for the first (R1) and second (R2) c-Myb repeats (see Additional File 1; data summarized in Table 1). However, because two adjacent acidic residues in both R1 and R2 are involved in salt bridge formation (asterisks in Additional File 1), it was difficult to evaluate the function of the acidic patch independent of the structural contributions of these residues. The third repeat has a single conserved acidic residue involved in a salt bridge (asterisks in Figure 2A) and disruption of this salt bridge not surprisingly led to loss of transcriptional activity (mR3-2 in Table 1). However, third repeat mutants with intact salt bridges but with mutation of the two or three adjacent acidic residues (mR3-1, mR3-3, Figure 2A) demonstrated a dramatic reduction of transcriptional activity to one-quarter and one-tenth that of wild-type c-Myb, respectively (Figure 2C; see also Additional File 1). Interestingly, the reduction in activity of the third repeat mutants appeared to correlate with the amount of negative charge eliminated. The mR3-3 mutant lost three negatively charged residues and displayed less activity than mR3-1 which was missing two charged residues. In order to further understand the non-structural function of the acidic patch, we therefore focused our analyses on these two mutants of the third repeat.

**Table 1:** Summary of c-Myb mutants.

						**% Reporter Activity**			
	Repeat-substitutions	Missing salt bridges	DNA-binding	Nuclear	Stability	c-Myb	c-MybVP	c-MybΔ	Endogenous target gene activation

Wt		0	+++	++	+	100	100	100	++

R1	1-1 AEDA	0	+++	++	+	220	120	168	nd

R1	1–2 AEAA	1	+++	++	+	45	100	120	+

R1	1–3 AADA	1	+++	++	+	98	77	83	+

R1	1–4 AAAA	2	+	++	+	10	20	47	+/-

R2	2-1 AED	0	+++	++	+	94	74	nd	nd

R2	2-2 AEA	1	--	+/-	+	> 1	> 1	nd	nd

R2	2–3 AAA	2	--	-	+/-	> 1	> 1	nd	-

R3	3-1 EAEA	0	+++	++	+	25	100	33	-

R3	3-2 EAAA	1	--	++	+/-	> 1	> 1	nd	-

R3	3-3 AAEA	0	+++	++	+	10	98	19	-

nd = not done.

Carboxy-terminal domains of c-Myb have been reported to negatively regulate transcriptional activation through intramolecular interactions with the DBD [27-30]. The conserved C-terminal domain also negatively regulates the c-Myb transcriptional activation domain, independent of interactions with the DNA-binding domain [28]. In order to isolate the DBD from effects of C-terminal sequences, we used a truncated c-Myb protein (c-MybΔ in Figure 2B) containing the DBD and c-Myb transcriptional activation domain but missing the C-terminal negative regulatory domain. The mR3-3 truncated protein had a defect in transcriptional activity similar to the full length mR3-3 protein when compared to their respective wild-type proteins, indicating that the C-terminal sequences do not contribute to the mR3-3 phenotype (Figure 2C).

Another concern was that mutation of the acidic residues may affect DNA-binding. We reasoned that if the c-Myb DBD could still bind to DNA in a sequence-specific fashion, then fusion of the c-Myb DBD to the strong VP16 activation domain would overcome the transcriptional defect of the mutant protein. Indeed, fusion of the mR3-3 mutant DBD to the VP16 activation domain rescued its transcriptional activity (Figure 2C). These data indicate that this acidic patch mutant of the c-Myb DBD is still capable of interacting with DNA *in vivo*.

Analysis of multiple western blots revealed no significant difference in the stability of these mutants relative to wild type c-Myb (Table 1; see also Additional File 1). Furthermore, transcriptional activity was measured at saturating levels of wild-type and mutant c-Myb. Therefore, the decreased activity of the mR3-1 and mR3-3 mutants is an intrinsic property of these proteins.

### Third repeat c-Myb acidic patch mutants localize to the nucleus and bind DNA in vitro

Previous experiments used deletion analysis to define two nuclear localization signals (NLS) in the DBD and in another region of the v-Myb protein [31]. We utilized a Myb-specific antibody to perform immunofluorescence of QT6 cells transfected with wild-type and mutant c-*Myb *expression vectors to determine if the acidic residues were important for nuclear localization. The wild-type and third repeat acidic patch mutants all localized to the nucleus, were excluded from the nucleolus, and appeared to concentrate in more euchromatic regions of DNA delineated by areas that stained less brightly by propidium iodide [see Additional File 2]. Replacement of the C-terminus by the VP16 activation domain or truncation of the C-terminus did not affect this localization (data not shown).

Since mutations were made in the DNA-binding domain, determining the ability of mutant proteins to bind DNA provides an essential and stringent control for structural stability. We expected disruption of salt bridges to interrupt DNA-binding. However, alanine mutants with intact salt bridges should maintain their ability to bind DNA since these mutations are clustered on the non-DNA-binding surface and are not predicted to affect structural stability. The observation that fusion to the VP16 activation domain rescues reporter gene activation by mR3-1 and mR3-3 was consistent with the ability of the mutant DNA binding domains to associate with DNA. In addition, we conducted electrophoretic mobility shift assays (EMSA) using a radiolabeled oligonucleotide containing a single high-affinity Myb-binding site with nuclear extracts containing either wild-type or mutant c-Myb VP16 fusion proteins. The c-Myb-VP16 fusions were less susceptible to proteolysis in lysates than the full length proteins, and therefore were ideal for these studies.

**Figure 3:**
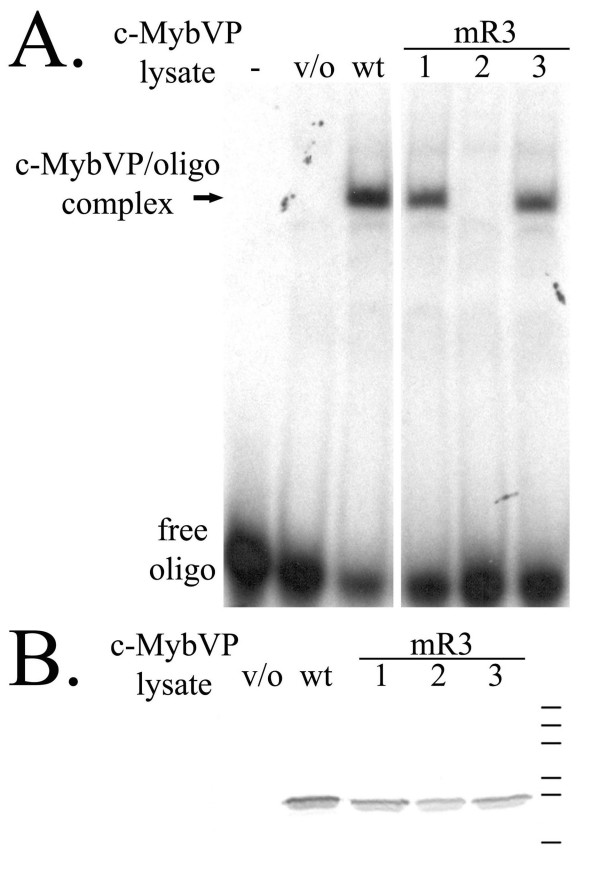
**Mutations in the third repeat bind DNA when the salt bridge is intact.** A. Electrophoretic mobility shift assays (EMSA) using nuclear lysates containing c-Myb VP16 (c-MybVP) wild-type and mutant fusion proteins were performed. c-MybVP lysates are labeled: – = no lysate; v/o = lysate with vector only (no c-MybVP protein); wt = wild-type; mR3-1,-2, and -3 = third repeat mutants. B. Western blot analysis of nuclear lysates used in the EMSA. The 5E Myb-specific monoclonal antibody reveals that similar wild-type and mutant proteins are present in the assay. c-MybVP lysates are labeled as in A. Bars represent the 7B SDS PAGE molecular weight markers (Sigma-Aldrich) with bands from top to bottom of 180, 116, 84, 58, 48.5, 36.5 kDa.

As expected, DNA-binding by the mutant proteins required intact salt bridges but was not disrupted by mutation of other acidic residues. In particular, mR3-1 and mR3-3 mutant proteins bound the oligonucleotide at levels similar to those of wild-type while disruption of the salt bridge in the mR3-2 mutant led to defective DNA-binding (Figure 3A). The DNA-binding activity of mutants in the first and second repeats was also dependent on the protein having intact salt bridges and is summarized in Table 1. western blot analysis indicated the presence of similar levels of wild-type and mutant proteins in these lysates (Figure 3B). In addition, similar affinities for specific DNA binding were observed with purified recombinant wild type and mutant c-Myb DBD produced in *E. coli *[see Additional File 3]. Together these results imply that the acidic residues in the third c-Myb repeat that are not involved in salt bridge formation have no effect on binding DNA *in vitro *or *in vivo*.

### The mR3-3 mutant is defective for activation of an endogenous target gene and cannot be rescued by the VP16 activation domain

**Figure 4:**
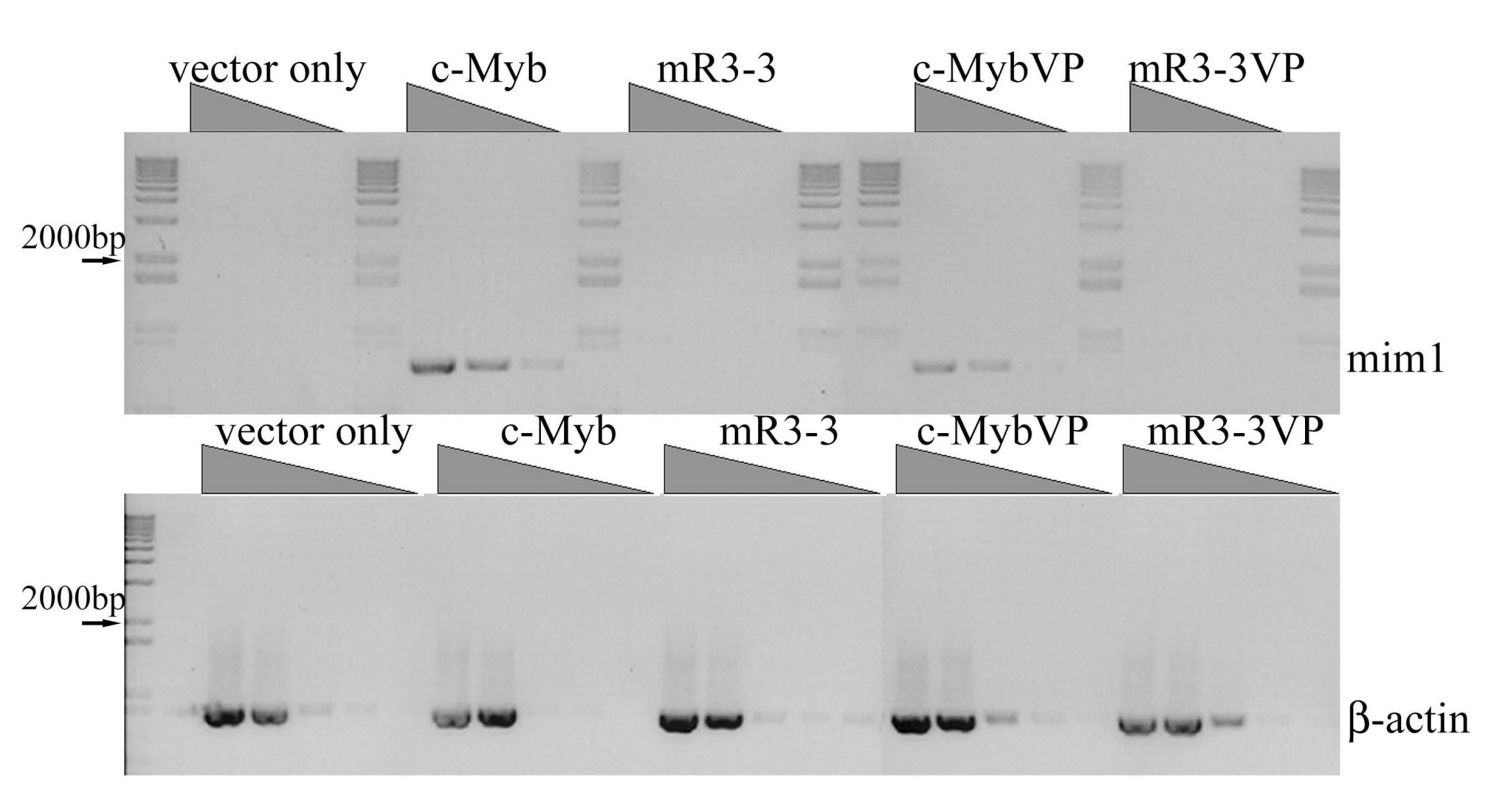
**The mR3-3 mutant is defective in activating the mim-1 endogenous target gene and cannot be rescued by the VP16 activation domain.** PCR was performed on ten fold serial dilutions of cDNA with primers specific for the mim-1 (676 bp) and β-actin (823 bp) genes. For mim-1 1:10^1;, 1:10^2;, and 1:10^3; dilutions are shown. For β-actin 1:10^2;, 1:10^3;, 1:10^4;, 1:10^5;, and 1:10^6; dilutions are shown. Expression of β-actin reveals that a similar amount of total RNA was used for each sample. Expression of mim-1 at a 1:10^3; dilution was detected for the full length wild-type c-Myb but no expression at any dilution was found for mR3-3. Complete absence of target gene activation was found for the mR3-3 DBD fused to VP16, while the wild-type c-MybVP did induce mim-1 expression. The 1 Kb Plus DNA molecular weight marker (Invitrogen) flanks each of the mim-1 samples and is at the left side of the β-actin gel. Sizes are 12,000 to 3000 bp for the top cluster of bands, then 2000 (arrows), 1650, 1000, 850, 650, and 500 bp for the lower bands.

The experiments described above measured transcriptional activation of transiently transfected reporter genes. However, previous studies have reported structural and functional differences between transiently transfected and genomic templates (reviewed in [32]). These differences may reflect inefficient chromatinization of transient templates as well as differences in regulation of supercoiling. Therefore, it was important to test the ability of our alanine mutants to activate expression of an endogenous target gene. We used RT-PCR analysis to determine whether the third repeat mutant could activate the expression of endogenous *mim-1*, a well-studied target of c-Myb whose activation depends on collaboration between c-Myb and C/EBPα [33]. Either wild type or mR3-3 mutant c-Myb was transfected into HD11 cells, macrophages which express endogenous C/EBPα but not c-Myb. We performed RT-PCR with specific primers to detect *mim-1 *expression and then normalized for total RNA using *β-actin *expression. RT-PCR on 10-fold serial dilutions of total RNA revealed that the wild-type c-Myb protein activated the endogenous *mim-1 *gene as expected, the product being detectable at a 1:1000 dilution of total RNA (Figure 4). However, activation of *mim-1 *expression by mR3-3 was undetectable at all dilutions tested. Thus, the acidic patch in the third repeat is essential for activation of this endogenous target.

Since fusion of the VP16 transcriptional activation domain rescued the ability of the mR3-3 mutant to activate a transfected reporter gene (Figure 2), we wondered if it would have a similar effect on endogenous gene activation. RT-PCR of total RNA from HD11 cells expressing wild-type or mR3-3 c-Myb-VP16 fusions revealed that the wild-type c-Myb-VP16 fusion could collaborate with C/EBPα to activate *mim-1 *expression, but the mR3-3 VP16 fusion could not activate transcription in the context of native chromatin (Figure 4).

### The ability of the mR3-3 mutant to interact with native chromatin is impaired

Since VP16 was able to rescue the mR3-3 transcriptional defect on a transiently transfected reporter but not on an endogenous gene, we suspected a defect in the ability of mR3-3 to associate with a fully chromatinized template. To test this hypothesis we performed protein chromatin immunoprecipitation (PChIP) assays that examine global association with chromatin by determining if a specific non-histone protein has a decreased capacity to pull down nucleosomes [see Additional File 4]. An advantage of this assay is that it provides a robust signal, allowing the experiment to be done without the use of stably expressing cell lines. Such experiments provide a more rigorous test of global chromatin association than assaying a single DNA site or a subset of sites, but cannot discriminate between differences on specific promoters.

Briefly, 293T cells were transfected with expression vectors for Flag-tagged wild-type c-Myb, mR3-3 c-Myb, or a vector only control. The cells were fixed with formaldehyde and cell lysates were sonicated to produce chromatin fragments of 100–300 bp in length [see Additional File 4]. Sonicated chromatin fragments were roughly the size of a single nucleosome; therefore, histones present in Myb-bound chromatin fragments represent the population of single nucleosomes bound by Myb. Anti-Flag-agarose beads were used to pull down Myb-bound chromatin fragments. Western blot analysis confirmed specific immunoprecipitation of Flag-c-Myb and Flag-mR3-3 at similar levels [see Additional File 4].

**Figure 5:**
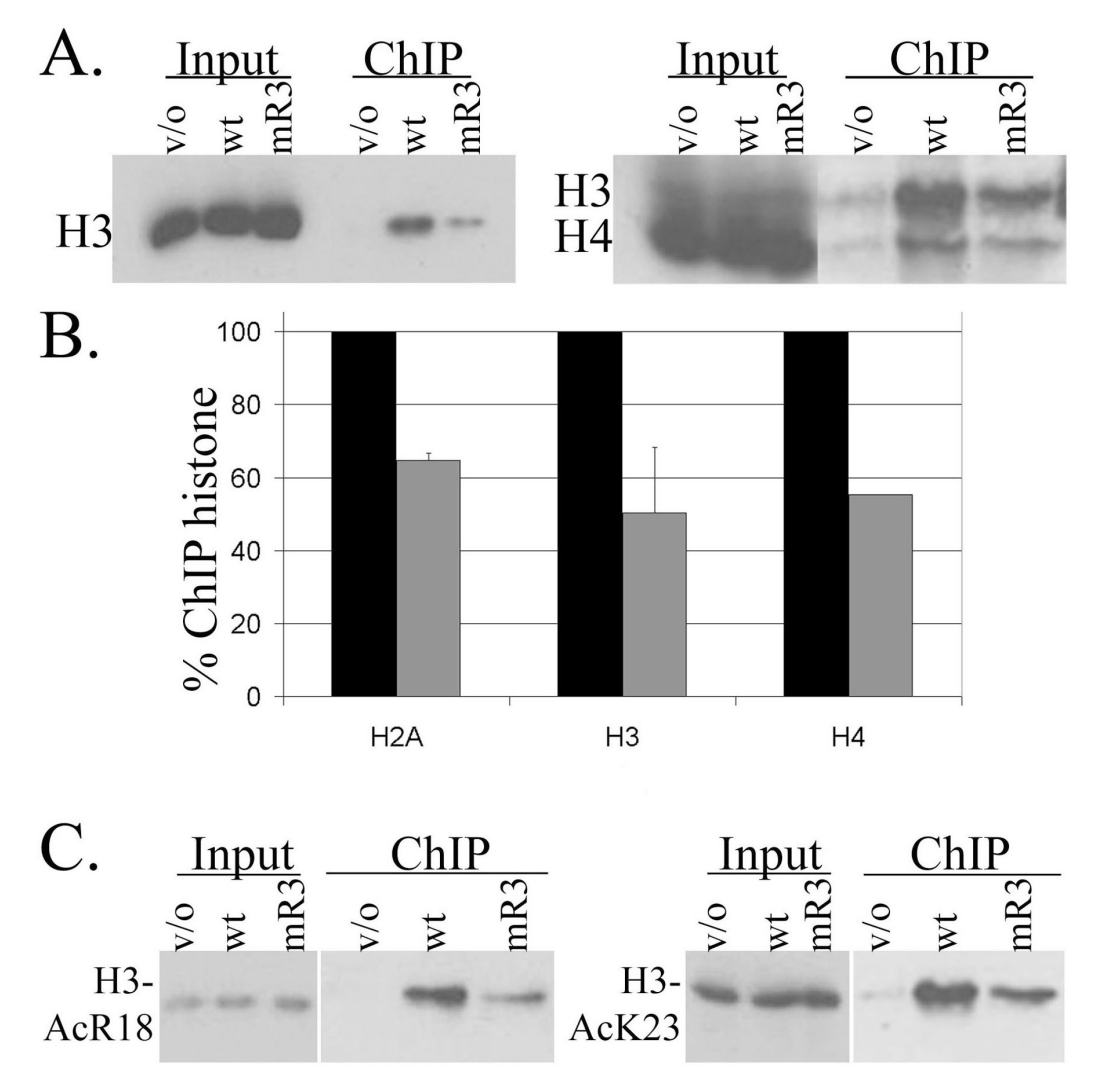
**The mR3-3 c-Myb mutant has impaired chromatin binding.** A. Western analysis was used to determine the amounts of core histones present in the vector only control (v/o), wild-type c-Myb (wt), and mR3-3 (mR3) chromatin immunoprecipitation fractions. Data displayed used antibodies directed at histone H3 (Abcam) and histone H4 (Upstate). Input and chromatin immunoprecipitation (ChIP) fractions are shown at different exposures. B. The relative amounts of core histones from several blots similar to those shown in (A) were quantitated and normalized for background using the ImageJ program . Wild-type levels were set to 100% and the mutants were normalized to this level for adequate comparison between different experiments. The plots shown represent an average of two separate experiments for H2A and five separate experiments for H3. The H4 antibody was used for only one experiment but is consistent with results for the other core histones. C. We compared the amount of H3-acetyl R18 (H3-AcR18) and H3-acetyl K23 (H3-AcK23) modifications present in the wild-type and mR3-3 bound nucleosomes using specific anti-H3-acetyl R18 and K23 antibodies (Upstate). Labeled as in A. Inputs are shown at a different exposure than chromatin immunoprecipitation (ChIP) samples and confirm that samples have similar amounts of input protein.

Since we suspected that the mR3-3 mutant had a defect in association with chromatin, we first looked at the fraction of core histones associated with mR3-3 compared to the wild-type c-Myb. Representative western blots are shown with quantitation of western blots from several lysates (Figure 5A and 5B). Quantitation of western-blots for H3, H4 and H2A showed a roughly 50% decrease in association of mR3-3 with core histones compared to wild-type c-Myb (Figure 5B). These results indicate a defect of the mR3-3 mutant proteins to bind native chromatin. Since this assay analyzes global chromatin binding one cannot differentiate between a partial defect of the mutant to associate with all binding sites and a complete defect in binding to half of the sites. Nevertheless, the fact that this effect is observed on a global chromatin fraction suggests that the acidic patch serves an important and global function in chromatin association by c-Myb.

Covalent modification of histones by acetyl, methyl, phosphate, ubiquitin, and sumo groups regulates nuclear processes including transcription, silencing, replication, and repair [34]. Work in this field supports a model in which these modifications provide a molecular blueprint, termed the histone code, for nuclear function at particular chromatin sites. For example, methylation of the N-terminal tail of histone H3 at lysine four (K4) is characteristic of active genes and the presence of euchromatin. A well-studied mark of heterochromatin is methylation at lysine nine (K9) of the histone H3 tail which provides a docking site for HP-1 (heterochromatin protein-1) and facilitates chromatin compaction. Thus, these two modifications provide a means of analyzing euchromatic and heterochromatic nuclear compartments. Analysis of wild-type c-Myb bound nucleosomes with antibodies to specific H3 modifications showed that both H3-dimethyl K4 (mainly euchromatin) and H3-dimethyl K9 (mainly heterochromatin) were present in wild-type c-Myb fractions (data not shown). Since the H3-dimethyl K9 modification can occur at lower levels in euchromatin, we confirmed our result with an antibody to H3-trimethyl K9 which localizes virtually exclusively to heterochromatin. The mR3-3 bound nucleosome fraction revealed reduced association with H3-dimethyl K4, dimethyl K9, and trimethyl K9 compared to wild-type c-Myb. The relative amount of modified histones in the mutant compared to wild-type c-Myb samples showed a reduction similar to that observed for the total core histones. These results indicate that the defect in association with chromatin of the mR3-3 c-Myb occurs in both the euchromatic and heterochromatic regions.

c-Myb directly binds to the CBP/p300 histone acetylase complexes leading to acetylation of histone H3 arginine eighteen (R18) and lysine twenty-three (K23) at c-Myb dependent promoters [35,36]. We found that nucleosomes marked by acetylation of acetylated H3-R18 and H3-K23 were bound 50–70% less by the mR3-3 mutant than by wild-type c-Myb protein, consistent with the binding results for total core histones (Figure 5C). Similar results were found with antibodies to H3-acetyl lysine nine (K9), lysine fourteen (K14), and H3-dimethyl arginine seventeen (R17) (data not shown). All of our results support a defect in global chromatin association for the mR3-3 mutant, indicating the acidic patch in the c-Myb R3 serves an important function in binding to native chromatin *in vivo*.

### Defective H4 tail binding by mR3-3 c-Myb mutant

**Figure 6:**
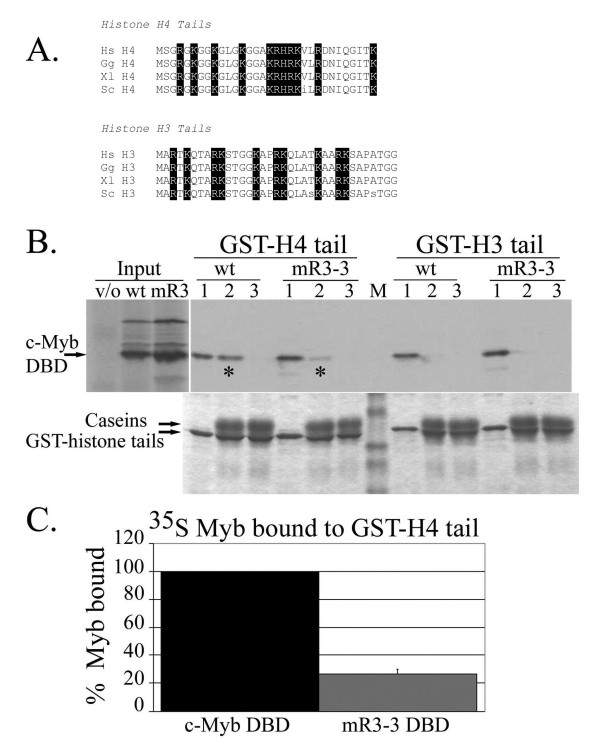
**The mR3-3 DBD demonstrates a selective defect in H4 tail binding.** A. Alignment of histone H3 and H4 tails is shown, with basic residues highlighted. Sequences are from H. sapiens (Hs), G. gallus (Gg), X. laevis (Xl), and S. cerevisiae (Sc). B. The ability of in vitro translated 35S-labeled c-Myb DBD to interact with GST-histone tail fusions (~25–27 kDa) was tested under three conditions: (1) NETN buffer; (2) GST binding buffer with low salt (50 mM KCl); (3) GST binding buffer with high salt (150 mM KCl). Similar amounts of wild-type (wt) and mR3-3 (mR3) 35S-labeled c-Myb DBD, shown with a vector only control (v/o), were used in each precipitation (different exposure than GST binding assays). Staining by Coomassie revealed a similar amount of GST-histone tails in each precipitation. A selective defect in the ability of mR3-3 to bind GST-H4 tail and be precipitated compared to wild-type was found under condition 2 (*). The broad bands directly above the GST-histone tails (conditions 2 and 3) reflects the use of nonfat milk and are likely the major casein proteins (~30–35 kDa). The Benchmark prestained protein ladder (Invitrogen) separates GST-H4 tail and GST-H3 tail (top to bottom are ~50, 40, 25, 20, and 15 kDa). C. Relative amount of the 35S-labeled c-Myb DBD bound to the GST-H4 tail was quantitated for two separate experiments using the ImageJ program . The wild-type (wt) c-Myb DBD was normalized to 100% and the mR3-3 (mR3) was scaled accordingly.

An impaired ability to bind to specific histone protein(s) rather than whole nucleosomes constitutes one possible explanation for the observed defect in chromatin binding. Therefore, we tested the ability of the wild-type and the mR3-3 mutant c-Myb DBD to bind histone tails. Association with histone tails makes biological sense for transcriptional regulators because histone tails extend out of the core nucleosomal particle, are essential for higher order chromatin folding, and are targets of many post-translational modifications [37,38]. Thus, opening of chromatin structure or reading of the histone code requires interaction with the histone amino termini. We used GST-yeast histone tail fusions in pull down assays with ^35^S-labeled *in vitro *translated c-Myb DBD [39]. Wild-type c-Myb activates transcription from integrated reporter genes in yeast, demonstrating that c-Myb is capable of interacting with yeast chromatin in vivo [25]. Furthermore, histones are highly conserved between yeast and vertebrates (Figure 6A; see also Additional File 5). A single conservative amino acid substitution exists between the yeast and human H4 tail (residue 22, Figure 6A) and the H3 tail has only two conservative changes (residues 23 and 32, Figure 6A). H2A and H2B are less conserved [see Additional File 5]. The H2A tail is 56% identical and 72% similar between yeast and human. The H2B tail is the most divergent between yeast and human, being only 41% identical and 50% similar 6.

The ability of wild-type and mR3-3 c-Myb to bind to GST-histone tails was tested under three different conditions: (1) those used in a previous study reporting a specific c-Myb association with the H3 tail [40]; (2) binding in the presence of glycerol, non-specific protein competitors, and a reducing agent in low salt (50 mM KCl); and (3) binding in the presence of glycerol, non-specific protein competitors, and a reducing agent plus a higher salt concentration (150 mM KCl). Under the first condition, we noted binding to all histone tails by both wild type and mR3-3 DBDs, with reduced binding to the H2A tail relative to other histone tails (Figure 6B; see also Additional File 5). Under the second condition which included non-specific competitor proteins, we observed binding of the wild-type c-Myb DBD to the H4 and H2B tails but not to the H3 or H2A tails (Figure 6B; see also Additional File 5]. These interactions could be disrupted with increased salt concentration in the third condition (Figure 6B; see also Additional File 5]. Remarkably, the mR3-3 DBD demonstrated an impaired ability to bind the H4 tail relative to the wild type DBD (80% reduction in the mutant). A similar reduction was not seen in binding to the H2B tail by mR3-3 versus wild type DBD. Thus, the mR3-3 DBD has a specific defect in binding the histone H4 tail relative to binding by the wild type DBD (Figure 6C).

## Discussion

Myb repeats were first discovered in the DNA-binding domains of proteins typified by the products of the v-*Myb *retroviral oncogene and its normal cellular progenitor, c-*Myb*. Such Myb domains adopt a helix-turn-helix fold found in many DNA-binding proteins in which basic residues interact with the negatively charged phosphate backbone of the DNA double helix. However, Myb-related domains are present in a wide variety of nuclear proteins, some of which do not themselves bind directly to DNA. We used phylogenetic analysis to identify a conserved acidic patch within the first of the three helices of most Myb domains. Remarkably, these acid patches lie on surfaces of DNA-binding Myb proteins that face away from DNA.

We have used alanine scanning mutagenesis to determine the function of the conserved acidic patches in the c-Myb protein. We chose to focus particularly on the acidic patch within the third Myb repeat of c-Myb because a mutant of this patch that did not affect protein stability via salt bridge formation, nuclear localization, or DNA binding, nevertheless was defective in transcriptional activation both of transfected reporter genes and of an endogenous target gene. We used biochemical analyses to demonstrate that the specific defect in transcriptional activation by this conserved acidic patch mutant correlated with a decrease in chromatin association *in vivo*. This mutant protein also displayed a reduced ability to interact with the histone H4 tail *in vitro*.

Although we observed a complete defect in activation of the endogenous target by the mR3-3 mutant, we detected only a 50% decrease in the ability of the third repeat mutant to bind chromatin *in vivo*. One possible explanation for our results is that c-Myb may utilize different methods of binding at distinct chromatin domains. The *in vitro *histone binding experiments demonstrated a specific defect of mR3-3 for binding the histone H4 tail but not the histone H2B tail. If the interaction of c-Myb with chromatin requires the H4 tail at a subset of sites and conversely the H2B tail at a different subset of sites, then the reduced ability to bind nucleosomes by mR3-3 in the chromatin immunoprecipitation assays would represent the loss of binding at the subset of sites requiring the c-Myb interaction with the H4 tail.

Another possibility is that all sites require both H4 and H2B tail association for stable c-Myb chromatin binding. In this case, the observed decrease in association with nucleosomes would represent a weakened interaction of the mR3-3 mutant with chromatin at all sites. Weakened association with nucleosomes would lead to a higher off-rate for the mutant protein bound to chromatin. However, the chemical crosslinking used in chromatin immunoprecipitation experiments might allow us to capture mutant protein bound to chromatin even if the mutant cannot occupy its chromatin site long enough to effectively activate transcription, thereby explaining the observed complete loss of activity in the context of a partial chromatin binding defect. The reduced association of the mR3-3 mutant with nucleosomes containing a variety of specific histone modifications was similar to that see for total core histones, supporting a global chromatin binding defect.

Although we observed a decreased binding of the mutant Myb protein to histone H4 tails *in vitro*, it is also possible that the defects in transcriptional activation and in chromatin association *in vivo *are due to interactions with non-histone proteins. For better or worse, the major histone proteins in metazoans are encoded by large clusters of tandemly repeated histone genes, making the generation and study of substitution mutants impossible with current technology. This is a general problem with all studies of covalent histone modifications and histone binding proteins. At present, the simplest model that explains all of our data is that the conserved acidic patch in Myb-related proteins is required for interaction with the basic region of the H4 tail, and that a loss of this interaction in the acidic patch mutant results in decreased chromatin occupancy which in turn results in a failure of transcriptional activation *in vivo*.

We note that genome-wide studies of chromatin occupancy by the sole Myb protein of Drosophila have shown that in a single cell line, greater than 30% of all promoters are occupied by a Myb protein complex [41]. One can hypothesize two classes of promoters: those which require the Myb acidic patch for initial access in their native chromatin environment; and those in which another protein creates an apparently nucleosome-free promoter region and thus do not require the Myb acidic patch for DNA occupancy by Myb protein. Such a model might explain why the VP16 activation domain can bypass the absence of the Myb domain acidic patch in poorly chromatinized templates for transcription typicial of transient transfection assays, whereas no such bypass occurs on the endogenous *mim-1 *gene in its native chromatin context.

Others have presented data arguing that the c-Myb but not the v-Myb DBD can specifically bind the histone H3 tail *in vitro *and have proposed that disruption of H3 tail binding by v-Myb is due to three mutations of hydrophobic residues in the second Myb repeat (N91I, H106L, D117V) [40]. Specific H3 tail binding by the c-Myb DBD was not observed in our experiments under several different conditions. However, by including competing proteins (1% nonfat dried milk), glycerol to disrupt hydrophobic interactions (10%), and a reducing agent (5 mM DTT), we did observe specific binding to the H4 and H2B tails, arguing that histone tail binding is an important function of the c-Myb DBD. Alanine mutagenesis of the acidic patch (mR3-3) led to a selective disruption of H4 tail binding. We did not see binding to the H3 tail by c-Myb in the presence of non-specific competitor proteins unless we used a concentration of GST-H3 tail ten-times higher than our standard conditions which did permit binding to H4 and H2B (data not shown).

The acidic patch of c-Myb was identified due to high conservation of these amino acids among the diverse group of Myb-related proteins, suggesting a common conserved function. Our results demonstrating chromatin and histone binding properties of the c-Myb DNA-binding domain are consistent with activities reported for other proteins containing Myb-related repeats. SANT domains represent a subset of Myb-related repeats in proteins that lack specific DNA-binding capacity [42]. Mutagenesis and deletion analysis of the Myb-related repeat, or SANT domain, in the ADA2 component of yeast GCN5 histone acetylase complex led to impaired histone acetylase activity and disrupted H3 tail binding of the complex [8,9]. Myb-related repeats in components of the ATP-dependent chromatin remodeling SWI/SNF and RSC complexes were also essential for *in vivo *activity [9].

The ISWI protein provides the enzymatic component of several ATP-dependent chromatin remodeling complexes and contains two Myb-related repeats. Nucleosome and histone H4 tail stimulated activity *in vitro *required the Myb-related repeats. One of the ISWI Myb-related repeats, termed the SLIDE domain, was also essential for stimulation of ISWI ATPase activity by free DNA, indicating this Myb-related repeat contacts DNA as well as histones in chromatin [11]. Interestingly, deletion of the Myb-related repeat in the TFIIIB-B" subunit of RNA polymerase III disrupted transcription on native chromatin *in vivo *but not on a naked DNA template *in vitro *[43]. Myb-related domains are also present in protein complexes that associate with and regulate inactive heterochromatin, suggesting that Myb-related motifs can function in the context of different chromatin domains [44,45].

Our data may reflect a predilection for histone H4 binding by complexes containing the three-repeat Myb proteins. Consistent with this idea is the finding that the Drosophila MMB complex (Myb-MuvB/dREAM) specifically interacts with unmodified histone H4 tails [46]. Histone H4 constitutes a prime target for complexes involved in global regulation of chromatin because it is the only histone without known variants [47]. In contrast, histones H3 and H2A have relatively abundant nonallelic variants, some of which define particular chromatin domains. Association of the Myb domain with histone H4 may reflect the ability of Myb-related proteins to bind chromatin despite the structural context since nucleosomes in all chromatin domains would carry the same invariant H4.

An intriguing group of Myb-related proteins in yeast may provide further insight into the role of Myb family protein in vertebrates. Yeast general regulatory factors (GRF) consist of a group of four proteins (ScRAP1, ScREB1, ScTBF1, ScABF1) identified due to their involvement in multiple nuclear processes including replication, transcription, silencing, and telomere maintenance [48-50]. The presence of many binding sites throughout the genome and the existence of Myb-related DNA-binding domains in three of these proteins (ScRAP1, ScREB1, ScTBF1) are common to this group [51,52]. Data suggest that GRFs have a common mechanism since the binding site of one GRF can substitute for another and swapping of protein domains leads to functional GRF chimeric proteins [51,53]. GRFs were first identified because *in vivo *binding led to a nucleosome free region at promoters, giving rise to the hypothesis that GRFs function by local opening of chromatin structure [50]. Recent studies have implicated the REB1 GRF in establishing "poised" chromatin domains at about 60% of budding yeast promoters via localization of the variant histone H2A.Z at the boundaries of a nucleosome free region [52,54-56]. Association with an invariant H4 tail might be required to facilitate this replication-independent exchange of H2A variants. GRFs also function by insulating chromatin domains, such as between silenced heterochromatin and genes primed for activation [48,49]. The ability to properly establish and regulate such chromatin domains is essential for genomic stability and the loss of any single yeast GRF leads to cell death, possibly due to genomic instability.

Besides the obvious presence of a Myb DNA-binding domain, the Myb family of proteins share many common properties with yeast GRFs. Myb proteins have a short consensus binding site (PyAAC^G^/_T_G) present throughout the genome [57]. Visualization of GFP-tagged Dm-Myb revealed many cytologically visible binding sites on polytene chromosomes [58]. In addition, genome-wide studies have recently shown that this Myb complex is present at greater than 30% of all promoters in a single cell type [41].

Our finding of a conserved acidic motif essential for the biological function and required for efficient chromatin and histone association support a role for Myb proteins in binding to and possibly regulating chromatin structure. Consistent with this are the observations that other Myb-related proteins utilize their Myb domains for chromatin regulation. The Myb-like domain in yeast GRFs has been implicated in genomic partitioning and regulation of chromatin domains [49]. The Myb-related domains in SANT proteins have been shown to be required for *in vivo *function and are known to serve a role in histone binding. The Myb family is required for the regulation of multiple genomic processes, is essential for genomic stability, and we have demonstrated that it contains a conserved acidic motif necessary for transcriptional activity and efficient chromatin association. Because this acidic patch is among the most conserved features common to all Myb-related domains, our findings support the idea that the primary conserved function of Myb-related domains may be histone-binding rather than DNA-binding. We postulate that the acidic patch of the Myb-related proteins may interact with the same basic region of the N-terminal histone H4 tail that was bound to an acidic patch created by the H2A/H2B core in the crystal structure of the nucleosome [37].

## Methods

### Identification of Myb repeats

Homologs of protein families previously reported to contain domains similar to the Myb-repeat were identified in *Homo sapiens*, *Mus musculus*, *Gallus gallus*, *Drosophila melanogaster, Caenorhabditis elegans*, *Dictyostelium discoideum*, *Zea mays*, *Arabidopsis thaliana*, *Saccharomyces cerevisiae*, and *Schizosaccharomyces pombe *using NCBI BLAST.

### Alignments

Myb-repeats of the following protein families were used for alignments: three-repeat transcription factors (Myb family, yeast BAS1), two-repeat transcription factors (plant Myb proteins), telobox, Swi3, Rsc8, ISWI, TFIIIB-B", NCor, Ada2, CDC5, REB1/TTF1, RAP1, CDC5, SNAPc4, and the arthropod R2 retrotransposon class. Highly divergent turns/loops between the helices were deleted to facilitate the alignment of the second and third helices. Identification and deletion of the turns/loops was facilitated by the Simple Modular Architectural Research Tool (SMART) program , but in some cases the length of the loop necessitated adjustment by hand [59]. These sequences were aligned using the ClustalW program [24].

The protein sequence for the N-terminal histone H4, H3, H2A, and H2B tails were retrieved from the PubMed database  for *Homo sapiens *(H. sapiens), *Gallus gallus *(G. gallus), *Xenopus laevis *(X. laevis), and *Saccharomyces cerevisia *(S. cerevisiae). These sequences were then aligned using the ClustalW program. H2A and H2B alignments were adjusted by hand to facilitate a better alignment of residues but a greater number of gaps. Shading and formatting of all alignments was done using BioEdit .

### Mutagenesis

The acidic residues in the first helix of each of the three repeats (R1, R2, and R3) were substituted with alanines using the Stratagene Quickchange kit (Figure 2a). Alanine residues were chosen because they have minimal effects on protein structure and disrupt interactions between charged surfaces [60]. Complementary primers extending approximately twenty nucleotides past both ends of the mutagenesis site were synthesized which substituted three to four nucleotides in close proximity to create the alanine substitutions. The primer sets used for mutation of the first repeat (R1) changed EEDE to AEDA, AEAA, AADA, and AAAA. The AEAA and AAAA mutations were monitored by creation of an AlwNI restriction site, while AEDA and AADA were monitored by DNA sequencing. Primers converting the second repeat (R2) residues EED to AED, AEA, and AAA led to disruption of a BseRI site. Mutation of the third repeat (R3) created another unique AlwNI site and changed the EEED to EAEA, EAAA, and AAEA. Mutagenesis was performed in the SP73-c-Myb plasmid and mutant DNA-binding domains were sequenced and recloned into the appropriate vectors for expression.

### Plasmids

All of the mutants were cloned into pcDNA3.1/myc-His-B mammalian expression vector (Invitrogen) as follows. Initially, an XbaI/HpaI fragment containing the c-MybVP16 fusion gene was subcloned from the N-CC-VP [61,62] vector into pcDNA3.1/myc-His-B using the XbaI/PmeI restriction sites. This strategy eliminated the myc-His tag from the final pcDNA3.1/c-MybVP16 product. Mutant c-Myb DBDs excised from the SP73-c-Myb vector using the unique Bsu36I and BstEII restriction sites were sub-cloned into pcDNA3.1-c-Myb-VP16 using the same restriction sites. Full length wild-type and mutant c-Myb expression vectors were made by replacing the BstEII/ClaI C-terminal coding fragment from the pcDNA3.1-cMyb-VP16 vectors with the BstEII/ClaI C-terminal coding fragment from the SP73-cMyb vector. The truncated c-Myb (Δc-Myb) was constructed by swapping the BstEII/ClaI fragment of the pcDNA3.1-cMyb-VP16 for the BstEII/ClaI fragment of the retroviral vector, N-I-CCd (N-I-c-MybΔ) [61,62]. To facilitate analysis of DNA binding, the three-repeats (3R) of the mutant DNA-binding domains were subcloned into the MT7-His6-R1R2R3-c-Myb-Tag bacterial expression vector using Bsu36I and BstE II restriction sites [62]. All subclones were screened by restriction site analysis and vectors were sequenced.

### Cell culture and DNA transfection

Quail QT6 fibroblasts were cultured at 37°C in 10% CO_2 _in Dulbecco's modified Eagle's medium (Cellgro) containing 5% fetal bovine serum (Gibco). QT6 cells were split into 10 cm (Falcon #3003) or 15 cm (Falcon #3025) treated tissue culture dishes and transfected a day later using the Fugene6 transfection reagent (Roche) according to the manufacture's instructions. Cells were harvested 36–48 hours after transfection for reporter assays and western blotting.

Chicken HD11 macrophages were cultured at 37°C in 10% CO_2 _in Dulbecco's modified Eagle's medium (Cellgro) containing 5% fetal bovine serum (Gibco) and 5% chicken serum (Sigma) on noncoated tissue culture plates (Falcon #1005). For transfections, cells were plated onto coated 10 cm (Falcon #3003) or 15 cm (Falcon #3025) dishes and allowed to grow overnight. Transfections were performed with Lipofectamine 2000 (Invitrogen) according to the manufacturer's instructions. Cells were grown for 24 hours in antibiotic-free media before harvesting.

### Reporter assays

Reporter assays were performed in QT6 cells by transfecting, 0.3 micrograms CMV-β-galactosidase, 0.1 micrograms CMV-EGFPc1 (Clonetech), 1.6 micrograms EW5-E1b-Luciferase reporter, and eighteen micrograms pcDNA3.1 c-Myb wild-type or mutant expression constructs [63]. The cells were harvested, lysed by freeze/thawing, and assayed for β-galactosidase and luciferase activity. The values obtained in relative luciferase units (RLU) were normalized for transfection efficiency using the β-galactosidase activity as an internal control. In order to easily compare experiments the normalized relative luciferase units were plotted as a percentage of the wild-type activity which was set to 100% activity. The results for each mutant are the average of three to six separate experiments. Truncated c-Myb had a transcriptional activity 100 times stronger than the full length c-Myb and the VP16 c-Myb fusion had an activity 1000 times greater than the full length. Thus, the wild-type full length, truncated, and VP16 fusion of c-Myb were all set to 100% and the respective mutants were scaled accordingly to facilitate comparison of the change in mutant activity relative to the corresponding wild-type protein.

### Electrophoretic mobility shift assays

For analysis of vertebrate cell extracts, QT6 fibroblasts were transfected in 10 cm dishes (Falcon #3003) using twenty micrograms pcDNA3.1-MybVP16 fusion plasmids and 0.1 micrograms CMV-EGFP-C1 (Clontech) to monitor transfection efficiency. QT6 cells were scraped into one milliliter of PBS and pelleted at 2500 g for ten minutes. Nuclear extracts were made according to standard protocols. To investigate DNA-binding in the absence of C-terminal sequences the wild-type and mutant c-Myb DNA-binding domains were expressed in *E. coli *using the MT7-His6-R1R2R3-c-Myb-Tag expression vectors [62].

Electrophoretic mobility shift assays using QT6 nuclear extracts were conducted with a 26 base pair oligonucleotide containing a single strong Myb binding site from the mim-1 promoter [62]. One-tenth (four microliters) of QT6 nuclear extracts were preincubated on ice for thirty minutes in DNA-binding buffer (10 mM Hepes pH7.6, 30 mM KCl, 1 mM DTT, 1 mM EDTA, 10 mM Ammonium Sulfate, 5% Glycerol, 5% Ficoll 400, 100 micrograms/milliliter BSA, and 0.5× EDTA-free Complete Protease Inhibitors (Roche)) and one hundred nanograms each of Salmon Sperm DNA, Calf Thymus DNA, and poly d(I-C). Ten femtograms of a ^32^P Klenow labeled 26 bp mim-1A oligonucleotide was added to the reaction and incubated for another thirty minutes on ice. The entire reaction was loaded onto a one millimeter thick 5% polyacrylamide gel made using 0.25× TBE buffer and a ratio of 80:1 acrylamide:bisacrylamide. The gel was run at ten volts/cm at 4°C in the same buffer. The gel was dried onto Hybond N+ membrane (Amersham Biosciences) to minimize loss of the oligonucleotide and then exposed to x-ray film (Kodak) for three hour to overnight.

### Western blot analysis

In order to monitor the wild-type and mutant protein expression in our reporter assays, half of the QT6 cells from the reporter assays were pelleted and resuspended in two hundred microliters of SDS-PAGE loading buffer. Boiled samples were analyzed using western blot analysis by standard techniques. The loading of protein samples was adjusted for transfection efficiency by using β-galactosidase to normalize the amount loaded or by co-staining the western blot for GFP using an anti-GFP mouse monoclonal antibody (Clontech). The c-Myb protein was detected using the 5E mouse monoclonal antibody (1:5000) using HRP [64]. For western blot analysis of c-MybVP16 protein in EMSA assays bands were visualized using alkaline phosphatase.

### Immunofluorescence

To determine the nuclear localization of mutant c-Myb proteins we utilized immunofluorescent staining. 200 ng of pcDNA3.1-c-Myb wild-type and mutant vectors with full length, c-Myb-VP16 fusions, or truncated c-Myb genes were transfected into 50–70% confluent QT6 cells grown in 8-well chamber slides using Fugene6 (Roche). After 36–48 hours QT6 fibroblasts were stained with a mix of monoclonal antibodies: Myb 2.2 (1:3000), Myb 2.7 (1:3000), and 5E (1:5000) [64,65]. A goat anti-mouse secondary coupled to Alexafluor was used to visualize Myb proteins (Molecular Probes). The cells were mounted in Vecta Shield mounting media containing propidium iodide to stain nuclei. The fibroblasts were imaged using a Nikon Eclipse E800 and a Spot camera system (Diagnostic Instruments).

### Analysis of endogenous mim-1 expression

Expression of the endogenous *mim-1 *gene was measured by preparing total RNA and performing reverse transcriptase-PCR analysis (RT-PCR). HD11 cells were transfected with twelve micrograms of pcDNA3.1-cMyb expression vectors and 0.1 ug CMV-EGFP-c1 as described above. The next day total RNA was isolated using the Trizol reagent according to the manufacturer's instructions (Invitrogen). Total RNA was analyzed by denaturing agarose gel electrophoresis, revealing minimal degradation. Two micrograms of total RNA was treated with DNase I (Invitrogen) for fifteen minutes. Synthesis of cDNA was performed using Oligo-dT primers and the First Strand Synthesis RT-PCR Kit (Invitrogen). Detection of the *mim-1 *and *β-actin *genes using gene specific primers and standard PCR reactions were performed on ten-fold serial dilutions of the cDNA templates. The relative amount of total RNA for each sample was determined using amplification curves of *β-actin *expression. The quantity of *mim-1 *expression was determined by normalizing to the amount of total RNA in each sample using the *β-actin *level as a standard.

### Protein chomatin immunoprecipitation assays

293T cells were grown in 150 mm × 25 mm treated tissue culture dishes (Falcon #3025) as described above. Cells from five 150 mm × 25 mm dishes were lightly trypsinized and then fixed with freshly prepared 1% buffered formaldehyde for fifteen minutes at room temperature in a 50 ml Falcon tube with constant shaking. The crosslinking reaction was stopped by adding glycine to a final concentration of 50 mM. Cells were pelleted at 3000 rpm for five minutes, washed once with ten milliters of 1× PBS, and transferred to Falcon tubes (Falcon 2096). Cell pellets were resuspended in one milliliter of ChIP lysis buffer (50 mM Hepes pH 7.6, 140 mM NaCl, 1 mM EDTA, 0.1% deoxycholate, 1% Triton X-100) and incubated on ice for ten minutes to lyse the cells. The cell lysate was then sonicated twelve times for twenty seconds each at a duty cycle of 50% and a power setting of two in a Branson sonicator using a 1/8" microtip. The cell lysates were incubated on ice for two minutes after each pulse. The lysate was precleared by spinning at 10,000 × g for fifteen minutes at 4°C.

The immunoprecipitation was performed by adding 55 microliters of a 50% slurry of Flag M2 antibody coupled to agarose beads (Sigma) plus 50 micrograms/milliliter salmon sperm DNA for two hours to overnight at room temperature or 4°C, respectively. The beads were pelleted and washed three times in low salt buffer (0.01% SDS, 1% Triton X-100, 1 mM EDTA, 20 mM Tris pH 8.0, 150 mM NaCl), three times in high salt buffer (0.1% SDS, 1% Triton X-100, 2 mM EDTA, 20 mM Tris pH 8.0, 500 mM NaCl), two times in LiCl/detergent buffer (50 mM TrisHCl pH8.0, 500 mM LiCl, 0.5% NP-40, and 0.5% deoxycholate) and two times in TE buffer (pH 8.0). Protein complexes were released by incubating twice at 100°C for fifteen minutes in SDS-PAGE loading buffer. Western analysis with antibodies to modified and core histones (Abcam and Upstate) were performed using a standard protocol.

### Histone binding assays

GST-yeast histone tails were expressed in bacteria and prepared according to standard procedures [39]. The c-Myb DBD was *in vitro *translated and ^35^S-methionine labeled in rabbit reticulocyte lysate (TnT-Promega). Roughly equal amounts of GST-histone tail, c-Myb DBD, and twenty microliters of a 50% slurry of glutathione-Sepharose were added to one milliliter of binding buffer. Binding reactions were conducted for two hours at room temperature and beads were washed six times with the same binding buffer. The beads were then boiled for ten minutes and the eluted proteins were loaded on 12% SDS PAGE. Protein gels were stained with Coomassie Blue to stain GST-histones then gels were dried and exposed to X-ray film to visualize ^35^S-labeled c-Myb DBD. Binding conditions were as follows: (1) NETN buffer (20 mM Tris-HCl pH 8.0, 1 mM EDTA, 50 mM NaCl, 0.5% NP40) [40]; (2) GST binding buffer (20 mM Hepes pH 7.9, 50 mM KCl, 2 mM EDTA, 0.1% NP40, 10% glycerol, 0.5% nonfat milk, 5 mM DTT); or (3) GST Binding Buffer with 150 mM KCl.

## Competing interests

The authors declare that they have no competing interests.

## Authors' contributions

ERK was involved in the design of the experiments, the execution of the experiments, and the writing of the manuscript. DK and CC were involved in the execution of the experiments. JSL was involved in the design of the experiments and the writing of the manuscript. All authors read and approved the final manuscript.

## Supplementary Material

Additional file 1 Alanine mutagenesis of the acidic patch in the first helix reveals a functional defect in transcriptional activation. Click here for file

Additional file 2 c-Myb proteins with mutant acidic patches in the third repeat localize to the nucleus. Click here for file

Additional file 3 DNA binding by purified bacterially expressed proteins. Click here for file

Additional file 4 Protein chromatin immunoprecipitation. Click here for file

Additional file 5 The wild-type and mR3-3 c-Myb bind the H2B tail but not the 2A tail. Click here for file

Additional file 6 Figure legends for additional files 1, 2, 3, 4, 5. Click here for file
