# Supplementary figures and images for: A conserved acidic patch in the Myb domain is required for activation of an endogenous target gene and for chromatin binding
Source: Mol Cancer. 2008 Oct 7;7:77. doi: 10.1186/1476-4598-7-77 (PMC2572630; doi:10.1186/1476-4598-7-77)

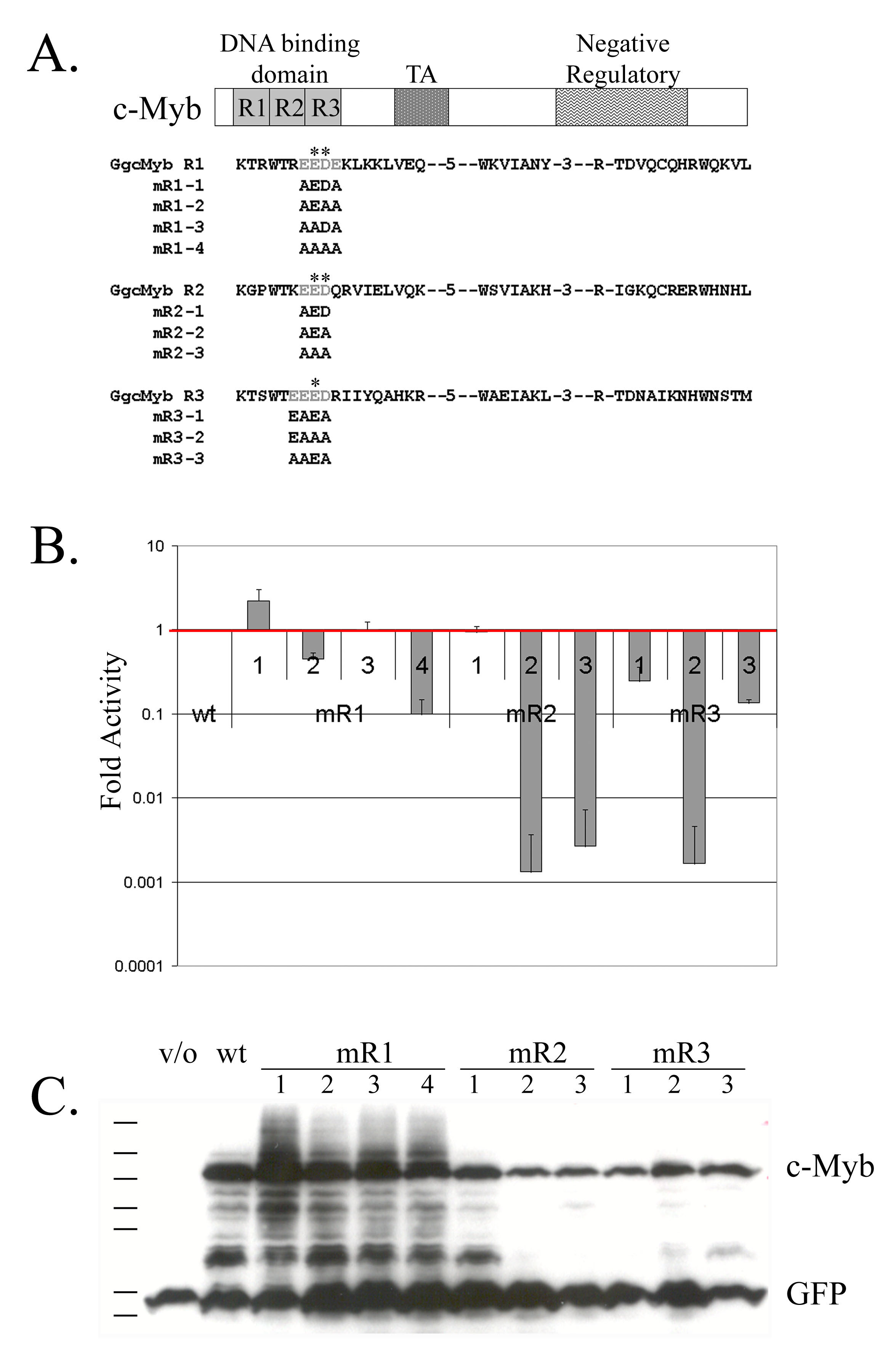

Supplement: Additional file 1 — Alanine mutagenesis of the acidic patch in the first helix reveals a functional defect in transcriptional activation. [file 1476-4598-7-77-S1.jpeg]

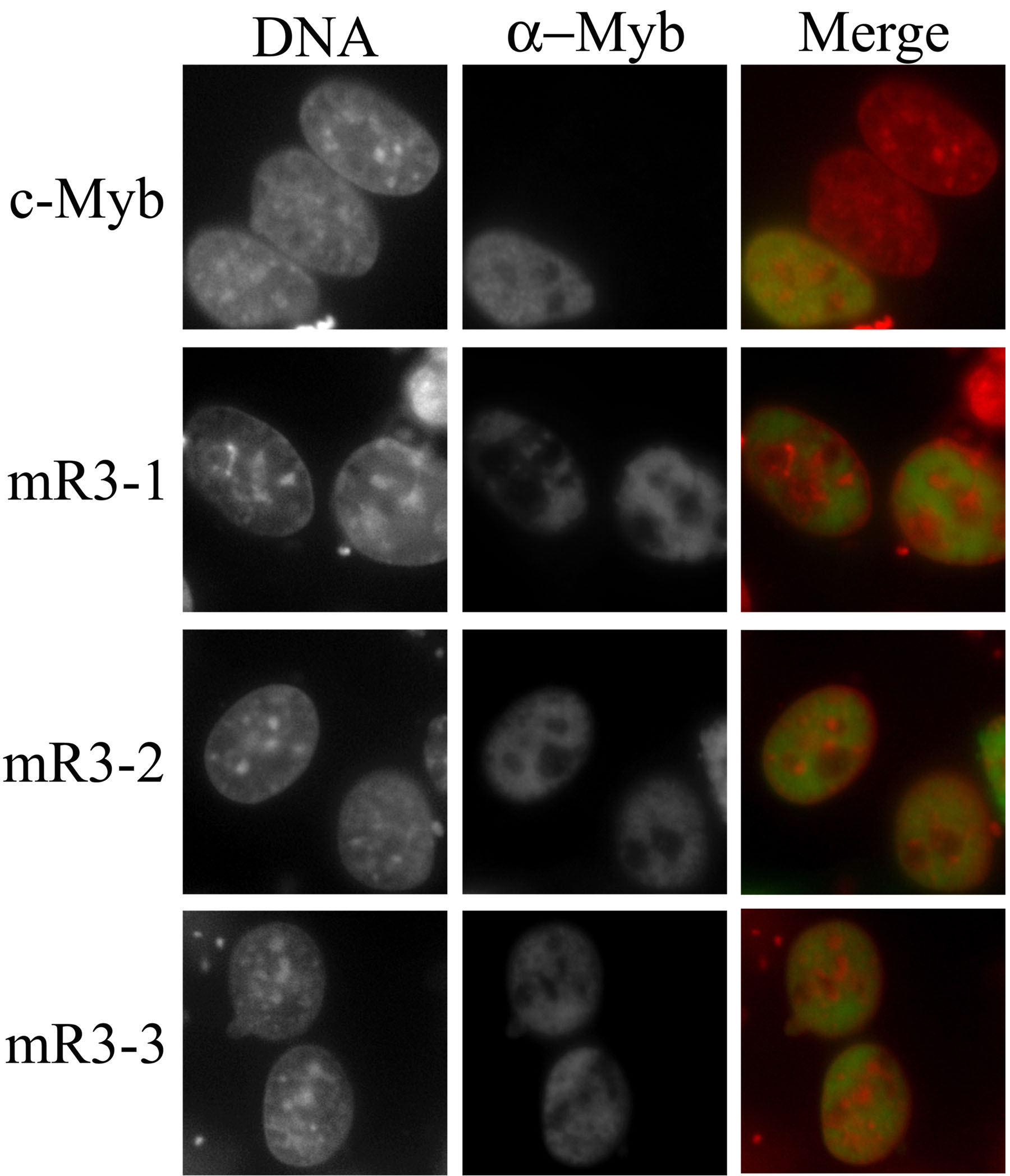

Supplement: Additional file 2 — c-Myb proteins with mutant acidic patches in the third repeat localize to the nucleus. [file 1476-4598-7-77-S2.jpeg]

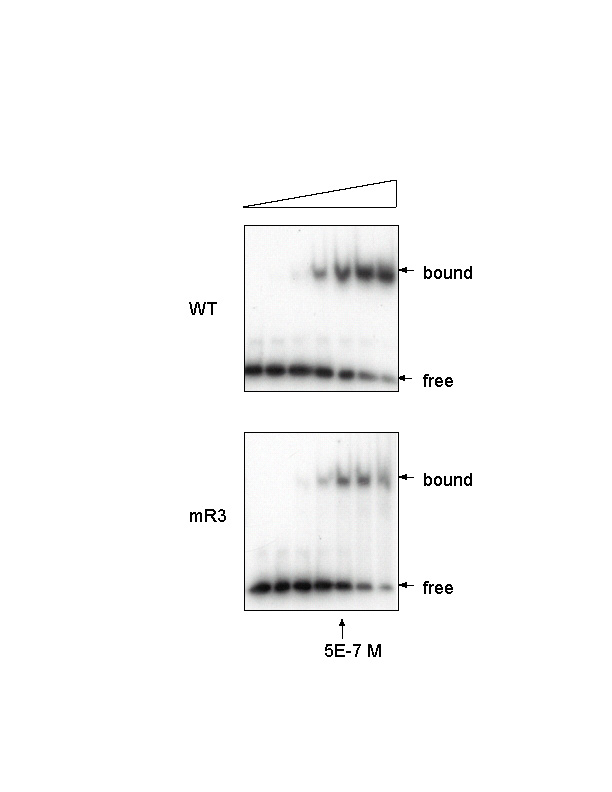

Supplement: Additional file 3 — DNA binding by purified bacterially expressed proteins. [file 1476-4598-7-77-S3.jpeg]

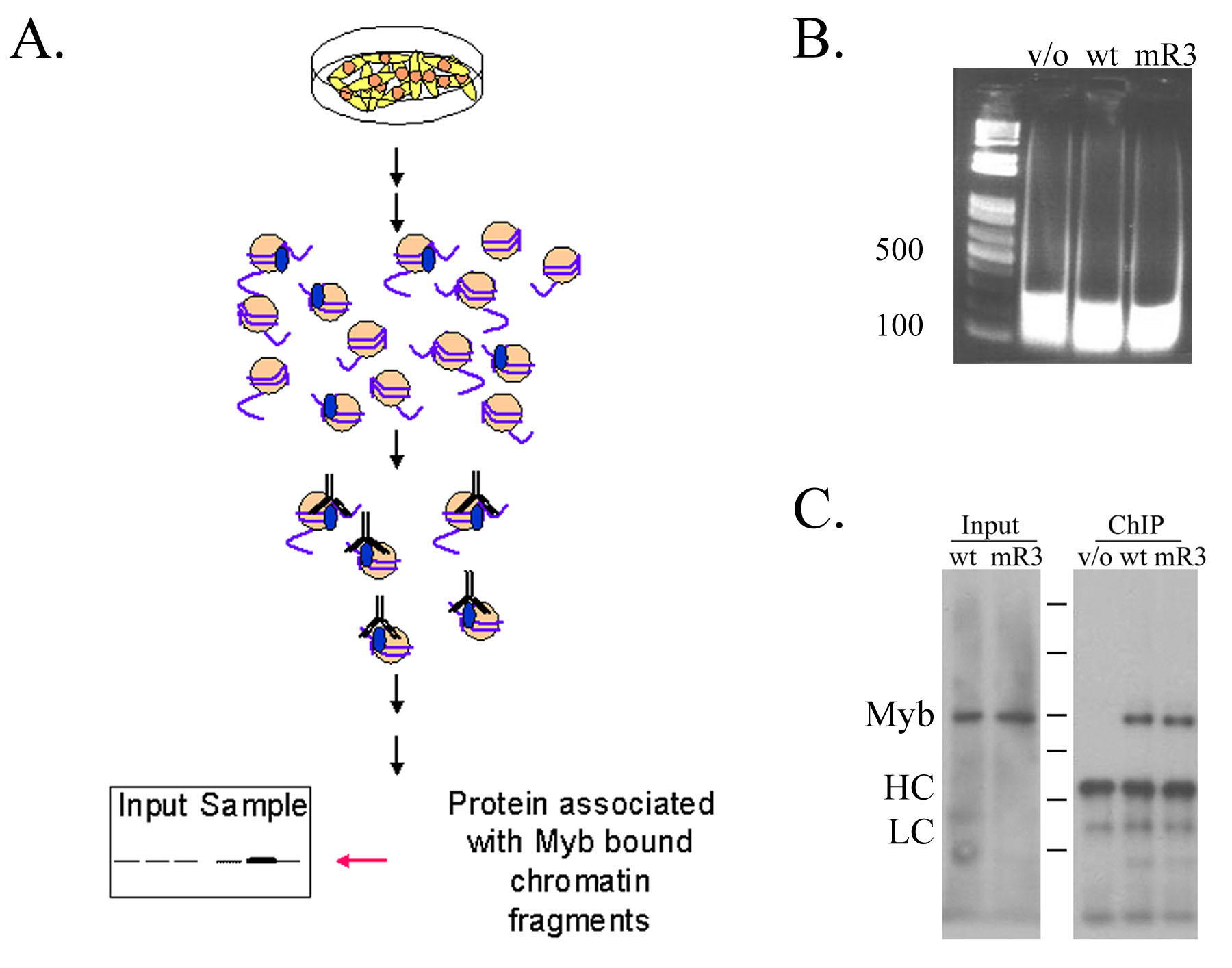

Supplement: Additional file 4 — Protein chromatin immunoprecipitation. [file 1476-4598-7-77-S4.jpeg]

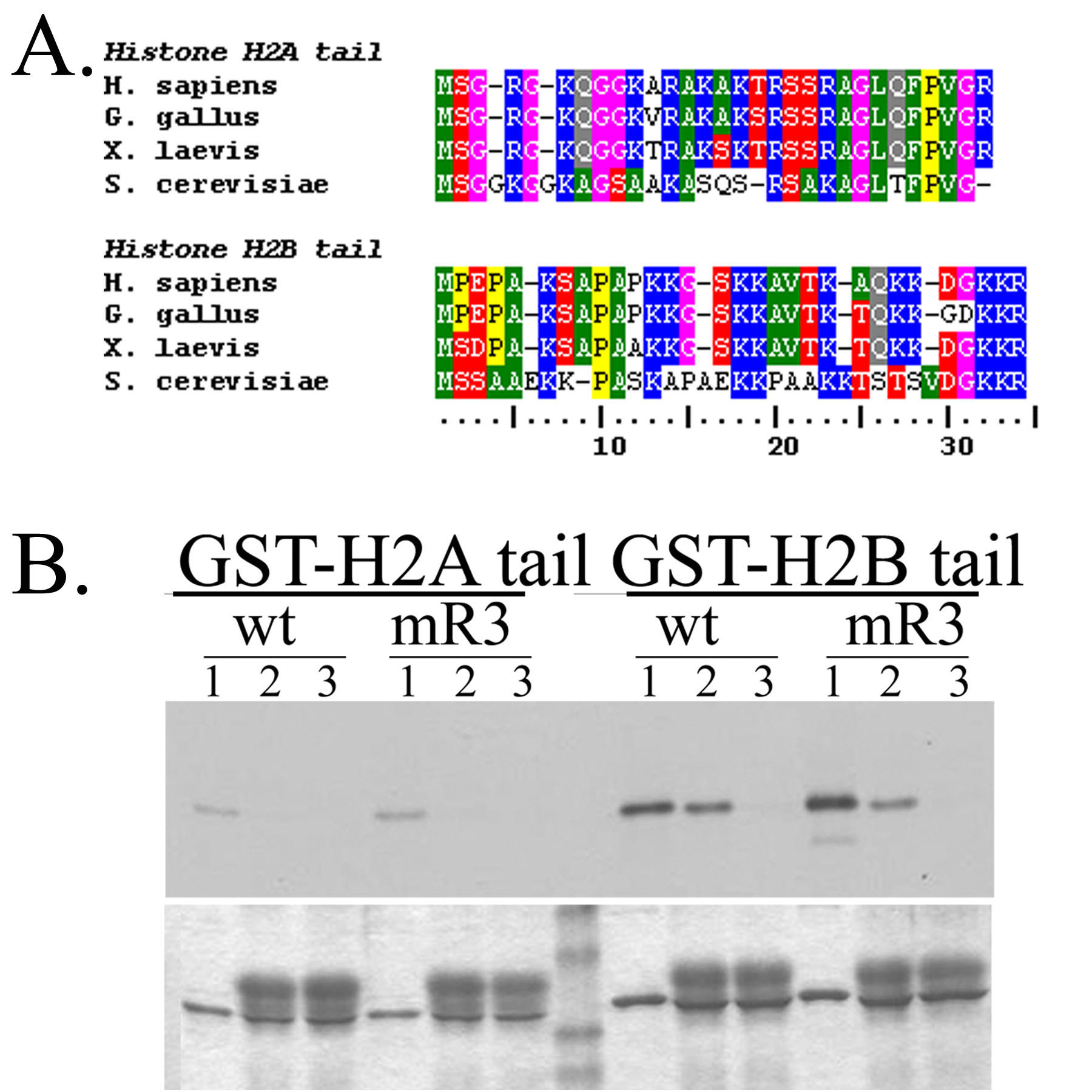

Supplement: Additional file 5 — The wild-type and mR3-3 c-Myb bind the H2B tail but not the 2A tail. [file 1476-4598-7-77-S5.jpeg]
