# Supplementary material for: A conserved acidic patch in the Myb domain is required for activation of an endogenous target gene and for chromatin binding
Source: Mol Cancer. 2008 Oct 7;7:77. doi: 10.1186/1476-4598-7-77 (PMC2572630; doi:10.1186/1476-4598-7-77)
Supplement: Additional file 6 — Figure legends for additional files 1, 2, 3, 4, 5. [file 1476-4598-7-77-S6.pdf]

Figure Legends for Additional Files 1-5

Additional File 1. Alanine mutagenesis of the acidic patch in the first helix reveals a functional defect in transcriptional activation. Alanine mutagenesis of c-Myb was performed to analyze the structural and functional contributions of the acidic patch in the first helix of each repeat. A diagram of the conserved domains of the full length protein is represented. The primary sequence of each of the three repeats is shown. Four first repeat mutants (mR1-1,2,3,4) were made: mR1-1 preserved both salt bridges, mR1-2 and mR1-3 preserved one salt bridge, and mR1-4 eliminated all acidic residues in the first repeat, including both salt bridges. Three mutants of the second repeat (mR2-1,2,3) were made: mR2-1 preserves both salt bridges, mR2-3 preserves the most highly conserved salt bridge, and mR2-3 eliminates all three acidic residues. Three mutants of the third repeat (mR3-1,2,3) were constructed: mR3-1 preserved the salt bridge, mR3-2 eliminated the salt bridge, and mR3-3 preserved the salt bridge and changed all three acidic residues not involved in salt bridges to alanines. Luciferase activity for each of the mutants was measured using the EW5-E1b-Luciferase reporter and was normalized for transfection efficiency by measuring β-galactosidase activity of a cotransfected CMV-β-gal plasmid. The wild type c-Myb values were then set to one (1=100% wild-type activity) and the mutants were scaled accordingly, allowing more accurate comparison of multiple experiments. A red line was placed at a value of one, bars above the line represent greater activity than wild type, bars below the line represent decreased activity compared to wild type. The data are presented on a log scale to normalize activation and repression. The fold activity for each mutant represents an average of three to six independent experiments. Error bars correspond to the standard deviation between experiments.

Western blot analysis for full length c-Myb and a cotransfected CMV-GFP was performed on lysates from the Luciferase assays. Cotranfected CMV-GFP acts as an internal control for transfection efficiency and as a loading control. The location of c-Myb and GFP protein bands are indicated. Bars specify the 7B SDS PAGE molecular weight marker (Sigma-Aldrich) with bands from top to bottom of 180, 116, 84, 58, 48.5, 36.5, 26.6 kDa. The 5E Myb-specific monoclonal antibody recognized Myb proteins and a mouse monoclonal GFP antibody recognized GFP.

Additional File 2. c-Myb proteins with mutant acidic patches in the third repeat localize to the nucleus. Immunofluorescence was performed on QT6 cells transfected with wild type or mutant full length c-Myb expression plasmids. Monochromatic images are shown for staining of DNA (propidium iodine) and Myb (5E/2.2/2.7 monoclonal antibody mix). The merged image depicts DNA in red and Myb in green. Note that not all cells were transfected in each field.

Additional File 3. DNA binding by purified bacterially expressed proteins. Wild type and mutant DNA-binding domains were expressed in *E coli* as soluble proteins with N-terminal His6 tags, and then purified to approximately 95% homogeneity by nickel chelate affinity chromatography. Equal amounts of each protein were serially diluted, then assayed for specific DNA-binding to a radioactive oligonucleotide containing the *mim-1A* site using the electrophoretic mobility shift assay (EMSA) in the presence of excess non-specific unlabelled competitor DNA as previously described (Dini and Lipsick, Mol Cell Biol 13: 7334-48, 1993). Very similar binding affinities were observed for wild type and mutant DNA-binding domains.

.

Additional File 4. Protein Chromatin Immunoprecipitation. The diagram depicts the steps of the protein chromatin immunoprecipitation assay. 293T cells were transfected with Flag-tagged wild type or mR3-3 full length c-Myb expression vectors. Protein-DNA complexes were fixed with 1% formaldehyde, cell lysates made, and sonicated to yield soluble chromatin fragments. Chromatin fragments were then immunoprecipitated using anti-Flag (M2) antibody coupled to agarose beads. After extensive washing, crosslinking was reversed and western blot analysis was used to determine proteins associated with c-Myb bound chromatin. Sonication of all samples yielded DNA fragments about 100-300bp in length.

Therefore, similarly sized chromatin fragments about the length of a single nucleosome were obtained for each sample.

Western blot analysis using the anti-Flag antibody revealed that similar levels of wild type and mutant protein were expressed in lysates. Importantly, similar levels of wild type and mR3-3 can be immunoprecipitated with the anti-Flag coupled beads. Antibody heavy chain (HC) and light chain (LC) were observed in immunoprecipitated samples as expected.

Additional File 5. The wild-type and mR3-3 c-Myb bind the H2B tail but not the 2A tail. We created multiple sequence alignments of histone H2B and H2A tails using ClustalW and color coded the alignments based on conservation using the BioEdit program. Multiple alignments included sequence from H. sapiens, G. gallus, X. laevis, and S. cerevisiae. The ability of in vitro translated ^{35}S-labeled c-Myb DBD to interact with GST-histone tail fusions (~25-27kDa) was tested under three conditions: (1) NETN buffer; (2) GST binding buffer with low salt (50mM KCl); (3) GST binding buffer with high salt (150mM KCl). Similar amounts of wild-type (wt) and mR3-3 (mR3) ^{35}S-labeled c-Myb DBD, shown with a vector only control (v/o), were used in each precipitation (different exposure than GST binding assays). Staining by Coomassie revealed a similar amount of GST-histone tails in each precipitation. Both the wild-type and mR3-3 proteins bound the H2B tail but not the H2A tail. The broad bands directly above the GST-histone tails (conditions 2 and 3) reflects the use of nonfat milk and are likely the major casein proteins (~30-35kDa). The Benchmark prestained protein ladder (Invitrogen) separates GST-H4 tail and GST-H3 tail (top to bottom are ~50, 40, 25, 20, and 15kDa).
